# Transforming growth factor β1 inhibition protects from noise-induced hearing loss

**DOI:** 10.3389/fnagi.2015.00032

**Published:** 2015-03-20

**Authors:** Silvia Murillo-Cuesta, Lourdes Rodríguez-de la Rosa, Julio Contreras, Adelaida M. Celaya, Guadalupe Camarero, Teresa Rivera, Isabel Varela-Nieto

**Affiliations:** ^1^Institute for Biomedical Research “Alberto Sols” (IIBM), Spanish National Research Council–Autonomous University of Madrid (CSIC-UAM)Madrid, Spain; ^2^Centre for Biomedical Network Research (CIBER), Institute of Health Carlos III (ISCIII)Madrid, Spain; ^3^Hospital La Paz Institute for Health Research (IdiPAZ)Madrid, Spain; ^4^Veterinary Faculty, Complutense University of MadridMadrid, Spain; ^5^Príncipe de Asturias University Hospital, University of Alcalá, Alcalá de HenaresMadrid, Spain

**Keywords:** cochlear injury, inflammation, noise-induced hearing loss, protection, TGF-β

## Abstract

Excessive exposure to noise damages the principal cochlear structures leading to hearing impairment. Inflammatory and immune responses are central mechanisms in cochlear defensive response to noise but, if unregulated, they contribute to inner ear damage and hearing loss. Transforming growth factor β (TGF-β) is a key regulator of both responses and high levels of this factor have been associated with cochlear injury in hearing loss animal models. To evaluate the potential of targeting TGF-β as a therapeutic strategy for preventing or ameliorating noise-induced hearing loss (NIHL), we studied the auditory function, cochlear morphology, gene expression and oxidative stress markers in mice exposed to noise and treated with TGF-β1 peptidic inhibitors P17 and P144, just before or immediately after noise insult. Our results indicate that systemic administration of both peptides significantly improved both the evolution of hearing thresholds and the degenerative changes induced by noise-exposure in lateral wall structures. Moreover, treatments ameliorated the inflammatory state and redox balance. These therapeutic effects were dose-dependent and more effective if the TGF-β1 inhibitors were administered prior to inducing the injury. In conclusion, inhibition of TGF-β1 actions with antagonistic peptides represents a new, promising therapeutic strategy for the prevention and repair of noise-induced cochlear damage.

## Introduction

Noise-induced hearing loss (NIHL) is the second most common form of deafness and constitutes an important public health priority. Animal studies have shown that exposure to excessive noise produces loss of hair cells, damage to the nerve synapses and loss of fibrocytes (Wang et al., 2002; Hirose and Liberman, 2003), leading to sensorineural deafness. The severity of hearing loss depends on noise characteristics (level, frequency, duration and temporal pattern) and genetic susceptibility (Ohlemiller and Gagnon, 2007).

The main underlying molecular mechanisms in NIHL include free-radical formation and oxidative stress, which activate cell death pathways in the cochlea, reduced cochlear blood flow, disruption of the blood-labyrinth barrier, glutamate excitotoxicity, calcium imbalance and cochlear inflammation (Henderson et al., 2006; Le Prell et al., 2007; Tan et al., 2013). Traditionally the cochlea was considered to be immunologically isolated because of the existence of the blood-labyrinth barrier. However, cochlear inflammation has been associated with many situations causing hearing loss, mainly otitis, autoimmune inner ear diseases and ototoxicity. In addition, in the last years inflammation has emerged as a key process in NIHL (Abi-Hachem et al., 2010; Tan et al., 2013).

Noise activates the local immune response, with early expression of proinflammatory cytokines in the cochlear resident macrophages (Okano et al., 2008), spiral ligament fibrocytes, strial cells and spiral ganglion neurons, including TNF-α, IL-1β and IL-6 (Ichimiya et al., 2000; Satoh et al., 2002; Fujioka et al., 2006; Tahera et al., 2006; Nakamoto et al., 2012; Tan et al., 2013; Zhang et al., 2013). These cytokines, along with other inflammatory mediators and cell adhesion molecules, induce the infiltration of blood monocytes and macrophages (Hirose et al., 2005; Tornabene et al., 2006) to phagocytize debris, and the secretion of more cytokines and growth factors (Yoshida et al., 1999). The inflammatory response is intended to limit the damage and promote further angiogenesis, fibroplasia and matrix synthesis, contributing to repair (Park and Barbul, 2004). However, it could also exacerbate pathological changes and produce bystander cell injury. Therefore, noise damaged cochlea represent a target for the application of otoprotective strategies based on controlling inflammation.

TGF-β is a member of a pluripotent cytokine superfamily with a key role in a variety of cellular processes such as proliferation, differentiation, extracellular matrix deposition and apoptosis during development, but also in postnatal stages (Massagué, 2012; Weiss and Attisano, 2013). In adult mammalians, TGF-β family members participate in the maintenance of tissue homeostasis, immune and inflammatory responses, angiogenesis and fibrogenesis (Dünker and Krieglstein, 2000; Prud’homme, 2007). The mammalian genome encodes for three isoforms (TGF-β 1, 2 and 3) with widespread tissue distribution and similar signaling cascades through TGF-β receptor types I, II and III (TGF-βR1, R2 and R3 or betaglycan) and SMAD2/3 proteins, which translocate to the nucleus and regulate gene transcription (Massagué, 2012). The three TGF-β isoforms are expressed in the embryonic cochlea in rodents with distinct patterns: TGF-β2 in the cochlear epithelium (Sanford et al., 1997; Paradies et al., 1998; Kim et al., 2006) and TGF-β1 and 3 in both epithelial and mesenchymal tissues (Pelton et al., 1991; Frenz et al., 1992; Paradies et al., 1998; Kim et al., 2006). Thus, TGF-β factors have been described to be involved in otic capsule formation (Liu et al., 2007), spiral ganglion formation and survival (Marzella et al., 1999; Okano et al., 2005), and indirectly in cochlear tonotopic organization (Son et al., 2012).

TGF-β1 is a master regulator of the immune response in several tissues, controlling the differentiation, proliferation, and activation of lymphocytes, macrophages and dendritic cells by autocrine and paracrine mechanisms (Letterio, 2000). Early after tissue damage, TGF-β1 modulates expression of adhesion molecules and induces chemoattraction and activation of leukocytes. These cells in turn secrete large amounts of interleukins, including TGF-β, which in a subsequent phase inhibit proliferation, differentiation and interleukin production (Letterio, 2000; Prud’homme, 2007; Mantel and Schmidt-Weber, 2011). Perturbations in the cytokine balance could modify dual TGF-β actions and contribute to immunopathology. In addition, several alterations of the immune system have been described in mice with targeted mutations in *Tgfb1*, including severe immune deregulation and lethal postnatal multi-organ inflammatory syndrome in *Tgfb1* knock-out mice (Shull et al., 1992; Kulkarni et al., 1993).

The role of TGF-β family factors in cochlear pathophysiology is not fully understood. Recent *in silico* analysis of genes relevant to hearing and deafness pointed to TGF-β1 as a nodal molecule in non-syndromic deafness and otic capsule development gene networks (Stamatiou and Stankovic, 2013). Although TGF-β1 is not among the classical proinflammatory cytokines, some studies in rodents have demonstrated an early increase in its expression during cochlear damage induced by aminoglycosides (Wissel et al., 2006), antigens (Satoh et al., 2006) and otitis media (Ghaheri et al., 2007), followed by a down-regulation as the response resolves, thus also supporting the immunomodulator role of TGF-β in the cochlea. Overexpression of TGF-β1 in the inner ear has also been related to fibrosis after cochlear damage (Kawamoto et al., 2003; Satoh et al., 2006), otosclerosis (Liu et al., 2007) and cochlear implantation trauma (Eshraghi et al., 2013). To our knowledge, there is no data concerning changes in TGF-β1 expression in NIHL, but a similar response to that observed in ototoxic or autoimmune labyrinthitis can be expected. Therefore, our hypothesis is that targeting TGF-β1 actions could help modulate the inflammatory response during noise-induced cochlear injury.

In this work we have studied the TGF-β signaling, gene expression and oxidative balance in the cochlea after noise exposure to clarify the role of TGF-β1 in NIHL. In addition, we have explored the potential of targeting this factor with two inhibitors of TGF-β1 as a therapeutic strategy to ameliorate the noise-induced functional, molecular and morphological changes.

## Materials and Methods

### Mouse Housing and Handling

Two month-old mice from three strains were used: C57BL/6JOlaHsd (C57), CBA/CaOlaHsd (CBA) and outbred HsdOla:MF1 (MF1) (Harlan Interfauna Ibérica, Spain). C57 mice are homozygous for a defective allele of the cadherin 23 gene (*Cdh23^ahl^*), and are especially vulnerable to noise (Ohlemiller et al., 2000; Park et al., 2013). In contrast, CBA is a normal hearing, noise injury-resistant strain (Wang et al., 2002; Ohlemiller and Gagnon, 2007). In our hands, MF1 background shows normal hearing and it is moderately resistant to NIHL (Celaya et al., in preparation).

Mice were fed *ad libitum* with a standard diet and drinking water, and controlled following FELASA recommendations. Animal experimentation was conducted in accordance with Spanish and European legislation and approved by the Spanish National Research Council (CSIC).

### Hearing Evaluation and Noise Exposure

Hearing was evaluated by registering the auditory brainstem response (ABR) as described (Cediel et al., 2006). Click and 8–40 kHz tone burst stimuli (0.1 and 5 ms duration, respectively) were generated with SigGenRP™ software (Tucker-Davis Technologies, Alachua, FL, USA) and presented monaurally at 30 or 50 pulses per second each, from 90 to 10 dBs relative to sound pressure level (dB SPL) in 5–10 dB SPL steps. The electrical response was amplified, recorded and averaged (1000 and 750 stimulus-evoked responses for click and tone burst, respectively). ABR thresholds were determined by visual detection and defined as the lowest intensity to elicit a reliable ABR wave with peaks I to IV clearly visible and medium peak amplitude over 200 nV. Peak and interpeak latencies were determined in the ABR trace in response to 20 dB SPL over the click evoked threshold.

The efficacy of TGF-β inhibitors was evaluated in a NIHL mice model. Briefly, conscious mice were confined in a wire mesh cage in the center of a reverberant chamber acoustically designed to reach maximum sound level with minimum deviation in the central exposure area (Cobo et al., 2009) and exposed to violet swept sine (VS) noise, at 100–120 dB SPL for short (30 min) or long (12 h) periods. VS noise was that was repeated during the 30 min of exposure.

VS noise was designed with Wavelab Lite software (Steinberg Media Technologies GmbH, Hamburg, Germany). It consists in a 10 s linear sweep in frequency, with a spectrum biased towards high frequencies (frequency range 2–20 kHz, VS^2–20^)and presented with a linear-with-frequency gain to compensate for the high frequency losses inside the chamber (Cobo et al., 2009; Sanz et al., 2015). The effect of noise exposure on hearing was evaluated with ABR tests as described above. The audiogram included tones from 8 to 40 kHz because exposure to noise of certain frequencies induces threshold shifts in octaves above those frequencies (Ou et al., 2000a,b; Sanz et al., 2015).

### Drug Administration

Two chemically synthesized TGF-β inhibitors (P17 and P144) with 95% purity were used in the study (DIGNA Biotech, Pamplona, Spain). P17 (KRIWFIPRSSWYERA) is a soluble hydrophilic peptide derived from a phage library (Dotor et al., 2007), with 100% relative binding affinity for TGF-β1, 80% for TGF-β2 and 30% for TGF-β3 (Gil-Guerrero et al., 2008). P144 (TSLDASIIWAMMQN) is a poorly soluble hydrophobic peptide derived from the sequence of the extracellular region of TGF-β type III receptor and specifically designed to block the interaction with TGF-β1 (Ezquerro et al., 2003).

Peptides stored at −80°C were gently defrosted, diluted in saline and sonicated (P144) until a clear solution was obtained. Peptides were administered intraperitoneally at doses ranging from 2.5 to 10 mg/kg, once or twice daily. These doses were shown to be effective in animal models of inflammation and fibrosis as mentioned before. Control mice were injected IP with a similar volume of saline (0.1 ml/10 g).

### Cochlear Morphology

At the end of every experiment, cochlear samples were taken for morphological evaluation. Cochlear samples were processed to obtain 5 μm thick paraffin sections following standard procedures. Cochlear morphology was studied in cresyl-violet (Fluka; Sigma Aldrich) stained sections with an Axiophot Zeiss microscope equipped with an Olympus DP70 digital camera as previously described (Riquelme et al., 2010).

### Gene Expression and Protein Analysis

When indicated, protein and gene expression were measured by Western blotting and reverse transcription coupled to quantitative PCR Data shown are representative of those found in the different mouse strains studied whenever there were no observable differences due to mouse background. For protein analysis, cochlear extracts were prepared as reported (Sanchez-Calderon et al., 2010). Protein concentration was determined using a Micro BCA Protein Assay Kit (Pierce Biotechnology, Inc., Rockford, IL USA) with BSA as the standard. Cochlear proteins were subjected to gel electrophoresis and transferred to PVDF membranes in a Bio-Rad Trans Blot apparatus. After incubation with a blocking solution, the membranes were probed overnight at 4°C with the following primary antibodies: p-SMAD2 (Ser465/467) (Cell Signaling Technology, Danvers, MA, USA), p-p38 MAPK (Thr180/Tyr182), NOX-4 (Santa Cruz Biotechnology, Santa Cruz, CA, USA) each at a 1:1000 dilution and MnSOD (Merck Millipore, Billerica, MA, USA; 1 μg/ml). Blots were re-probed with SMAD2/3 (Santa Cruz Biotechnology, 1:1000), p38α (Santa Cruz Biotechnology, 1:1000), β-actin (Sigma-Aldrich Corp. St. Louis, MO, USA; 1:2500) or p44/42 MAPK (Cell Signaling Technology, 1:1000) as loading controls. Antibodies were prepared in TTBS containing 5% BSA for phosphorylation-specific antibodies or non-fat dried milk for others. The membranes were washed and incubated with the corresponding peroxidase-conjugated secondary antibodies for 1 h at room temperature. Immunoreactive bands were visualized by enhanced chemiluminescence (GE Healthcare Bio-Sciences, Pittsburgh, PA, USA) using X-ray films (Agfa, Mortsel, Belgium), and the bands were quantified by densitometry with NIH ImageJ software. Different exposure times were used to ensure that bands were not saturated.

For gene expression studies, cochlear samples were processed for RT-qPCR as reported (Rodríguez-de la Rosa et al., 2014). TaqMan MGB probes were obtained from Assay-by-Design^SM^ (Applied Biosystems) for amplification of *Tgfb1, Tgfb2, TgfbR1* and *TgfbR2* genes. The ribosomal phosphoprotein P0 (*Rplp0*) was used as endogenous control gene. The relative quantification values (RQ) between noise exposed and non-exposed mice were determined by the 2^−ΔΔ^Ct method as reported (Sanchez-Calderon et al., 2010), where ΔΔCt = ΔCt_exposed_ − ΔCt_non-exposed_, and ΔCt = Ct_target_ − Ct_endogenous_. Data were expressed as log10RQ mean.

### Statistical Analysis

For ABR and Western blot results, statistical analysis was performed using IBM SPSS software (v.19.0). RT-qPCR data were analyzed using the Integromics Real Time StatMiner software package.^1^ Data were expressed as mean ± standard error (SEM), and the results were considered significant at *p* ≤ 0.05. Statistical significance was estimated by different tests for ABR, western blot and qRT-PCR data, which are specified in the figure legends.

## Results

### Noise Exposure Induces Changes in TGF-β Signaling, Gene Expression and Oxidative Balance in the Cochlea

**Figure 1:**
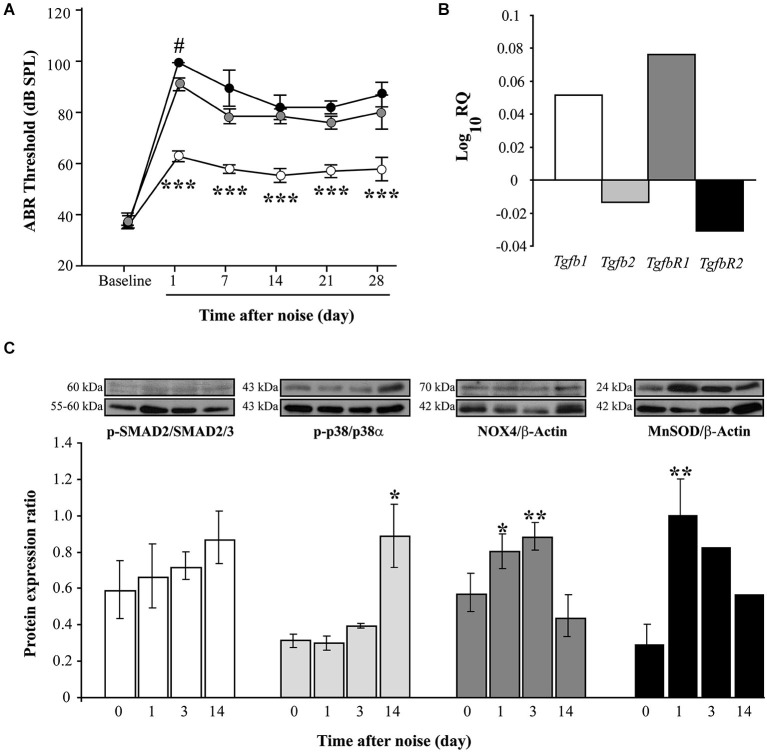
**Functional and molecular effects of noise exposure. (A)** Evolution of ABR click thresholds (mean ± SEM, in dB SPL) in C57 mice exposed to VS^2–20^ noise at 100 (white circles), 110 (gray) or 120 (black) dB SPL (*n* = 6 per group) for 30 min. Statistically significant differences were found between mice exposed at 100 dB SPL and the other groups (paired *T* test, ***p ≤ 0.001) and between 110 and 120 groups 1 day after exposure (#*p* ≤ 0.05). **(B)** Log_10RQ bar plot for *Tgb1*, T*gfb2, TgfbR1* and *TgfbR2* of cochlear samples, comparing noise exposed (VS^2–20^ at 110 dB SPL) with non-exposed CBA mice. Increase in the expression of *Tgfb1* and *TgfbR1* was observed in noise exposed mice compared to non-exposed, the latter with a statistical signifficant difference (*p* = 0.008, Limma test with Benjamini-Hochberg FDR method and 0.05 adjusted *p*-value threshold). **(C)** Time course of p-p38/p38α, p-SMAD2/SMAD2/3, NOX-4/β-actin and MnSOD/β-actin protein expression ratios in the cochlea of noise-exposed C57 mice (VS^2–20^ at 110 dB SPL), before and 1, 3 and 14 days after noise exposure (*n* = 3 mice for each determination). Representative blots are shown. Data shown as mean ± SEM. Statistically significant differences were found in p-p38, NOX4 and MnSOD levels after noise exposure compared to baseline values (paired *T* test, *p ≤ 0.05; **p ≤ 0.01).

Exposure to high level (100–120 dB SPL) VS^2–20^ noise induced a notably temporal threshold shift in the first 24 h after exposure and a gradual but limited recovery of ABR thresholds. The severity of hearing loss is related to noise intensity, with VS^2–20^ noise at 110 or 120 dB SPL inducing permanent changes that suggest irreversible cochlear damage (Figure 1A).

Next, the expression of *Tgfb* related genes (*Tgfb1, Tgfb2, TgfbR1* and *TgfbR2*) was studied in cochlear samples that were taken 4 and 24 h after challenge with VS^2–20^ noise at 110 dB SPL for 30 min. In general, RT-qPCR expression profiles of *Tgfb* related genes in noise exposed mice showed homogeneous distribution of Ct values and a higher correlation Pearson index compared to non-exposed controls. A statistical significant increase in the cochlear expression of *Tgfb1* was observed in mice exposed to noise 4 h after damage compared to non-exposed controls, (RQ value of 3.2 ± 0.5, *p* = 0.03). One day after exposure, a simultaneous increase in *Tgfb1* and *TgfbR1* and a decrease in *Tgfb2* and *TgfbR2* gene expression was observed in exposed mice compared to controls, although the change was statistically significant only for *TgfbR1* (RQ value of 1.2 ± 0.07, *p* = 0.008) (Figure 1B). In addition, p-SMAD2 protein levels, which propagate de TGF-β1 signal, as well as p-p38α, NOX-4 and MnSOD, which are related to cellular stress and oxidation, were determined prior to and 1, 3 and 14 days after noise exposure. Our results indicate a moderate and progressive increase in the phosphorylation of SMAD2 (ratio p-SMAD2/SMAD2/3) after noise exposure, compared to baseline values, although without statistically significant differences (Figure 1C). In addition, the levels of other stress-related molecules such as phospho-p38α, NOX-4 and MnSOD were also elevated in cochleae after noise damage, confirming the role of oxidative stress in the pathophysiology of NIHL. A statistically significant increase in the levels of the antioxidant enzyme MnSOD was observed in the first 24 h after exposure, whereas phosphorylation of p-38α MAPK rose significantly 2 weeks after injury. No differences were observed due to mouse strain (data not shown).

These results confirm that inflammation and oxidative stress are key underlying mechanisms in NIHL and show that TGF-β1 plays a role in cochlear response to noise injury. In this context, the use of TGF-β1 inhibitors could represent a therapeutic strategy for ameliorating NIHL. To evaluate the safety profile and the efficacy of TGF-β1 inhibitor peptides P17 and P144 in the prevention and treatment of NIHL, different experiments were carried out and they are summarized in Table 1.

**Table 1:** **Summary of the experiments performed with TGF-β1 inhibitors**.

Study	Strain	Drug	Dosage	VS^2–20^ noise level/duration
Safety	MF1, C57	P17, P144	2.5 mg/kg/24 h/15 days	-
Pretreatment	MF1, C57	P17, P144	2.5 mg/kg/24 h /15 days	100 dB SPL/12 h
Treatment	C57	P17, P144	2.5 mg/kg/24 h /30 days	100 dB SPL /12 h
		P17, P144	2.5 mg/kg/12 h/15 days	100 dB SPL /30 min
		P17, P144	2.5 mg/kg/12 h/15 days	110 dB SPL /30 min
		P17, P144	2.5 mg/kg/12 h/15 days	120 dB SPL /30 min
	CBA	P144	10 mg/kg/24 h/ 15 days	110 dB SPL /30 min

*Different mouse strains and doses were used to study cochlear toxicity and efficacy of TGF-β1 inhibitor peptides P17 and P144 in the treatment of NIHL. MF1, HsdOla:MF1; C57, C57BL/6JOlaHsd; CBA, CBA/CaOlaHsd; IP, intraperitoneal; VS^2–20^, Violet Swept Sine noise, frequency range 2–20 kHz*.

### TGF-β1 Inhibitors Reduce Functional and Morphological Alterations After Noise-Exposure

#### Safety Assessment of P17 and P144

Initially, the safety of treatment with TGF-β1 inhibitors was evaluated. Systemic administration of either P17 or P144 peptides at doses ranging from 2.5 to 10 mg/kg/24 h for 15 days in MF1 and C57 mice was well-tolerated. None of the treated mice showed signs of systemic toxicity nor did they die, confirming previous reports in mice and rats (Ezquerro et al., 2003; Arribillaga et al., 2011; Baltanás et al., 2013). With regard to hearing, treatment with P17 and P144 did not increase ABR thresholds or peak latencies, and no significant differences were observed between drug-treated and saline control mice (Table 2). In addition, cochlear samples were taken after treatments to evaluate inner ear morphology. No gross alterations were found in the organ of Corti, spiral ganglion and stria vascularis, confirming the non-ototoxic and safe profile of both peptides (data not shown).

**Table 2:** **Analysis of hearing parameters after treatment with TGF-β1 inhibitors**.

Strain	Group	ABR Parameter	*n*	Baseline		After treatment	
				Mean	SEM	Mean	SEM
	SSF	Threshold		41	1.79	42	2.68
		IPL I-II		1.03	0.04	1.03	0.01
		IPL I-IV	5	2.63	0.03	2.57	0.03
		IPL II-IV		1.60	0.05	1.54	0.04
		AMP I		1260.22	178.17	1231.64	177.30
MF1	P17	Threshold		39	2.45	36	2.04
		IPL I-II		0.93	0.02	0.90	0.02
		IPL I-IV	6	2.66	0.03	2.50	0.01
		IPL II-IV		1.73	0.03	1.60	0.03
		AMP I		1184.65	206.14	1488.37	220.30
	P144	Threshold		36	0.82	34	0.82
		IPL I-II		0.92	0.01	0.90	0.01
		IPL I-IV	6	2.65	0.04	2.47	0.03
		IPL II-IV		1.73	0.03	1.57	0.02
		AMP I		1191.12	106.64	1411.01	115.87
	SSF	Threshold		43	1.22	39	1.63
		IPL I-II		1.01	0.02	0.98	0.02
		IPL I-IV	6	2.93	0.05	2.56	0.05
		IPL II-IV		1.92	0.04	1.58	0.05
		AMP I		1232.68	96.29	813.64	67.12
C57	P17	Threshold		43	1.22	42	1.63
		IPL I-II		0.91	0.04	0.91	0.01
		IPL I-IV	6	2.66	0.10	2.53	0.05
		IPL II-IV		1.75	0.07	1.62	0.05
		AMP I		974.21	127.11	730.55	98.24
	P144	Threshold		41	1.63	40	1.22
		IPL I-II		1.01	0.01	0.95	0.03
		IPL I-IV	6	2.82	0.05	2.61	0.04
		IPL II-IV		1.82	0.05	1.67	0.03
		AMP I		1075.09	155.36	778.55	59.05

*ABR thresholds (in dB SPL), interpeak latencies (in ms) and peak I amplitude (in nV) in response to click stimulus before and after systemic (intraperitoneal) treatment with TGF-β1 inhibitor peptides P17 and P144 (at 2.5 mg/kg/24 h) or saline (at 0.1 ml/10 g/24 h) for 15 days in MF1 and C57 mice. No statistically significant differences were found between baseline and post-treatment values (paired T-tests). MF1, HsdOla:MF1; C57, C57BL/6JOlaHsd IPL, interpeak latency; AMP, peak amplitude*.

#### Pretreatment with P17 and P144 Reduces Threshold Shift After Noise Exposure

The potential of TGF-β1 inhibitors in preventing NIHL was evaluated by treating mice with P17 and P144 at 2.5 mg/kg/24 h or with saline at 0.1 ml/10 g/24 h for 15 days and then exposing them to VS^2–20^ noise at 100 dB SPL. ABR was performed before the treatment, and 1 day, 1, 2, 3 and 4 weeks after noise exposure. The experimental procedure was performed on mice from two genetic backgrounds, MF1 and C57, with different susceptibilities to noise damage. Under all the conditions studied, click thresholds did not change after treatment with P17 or P144, confirming the safety profile.

**Figure 2:**
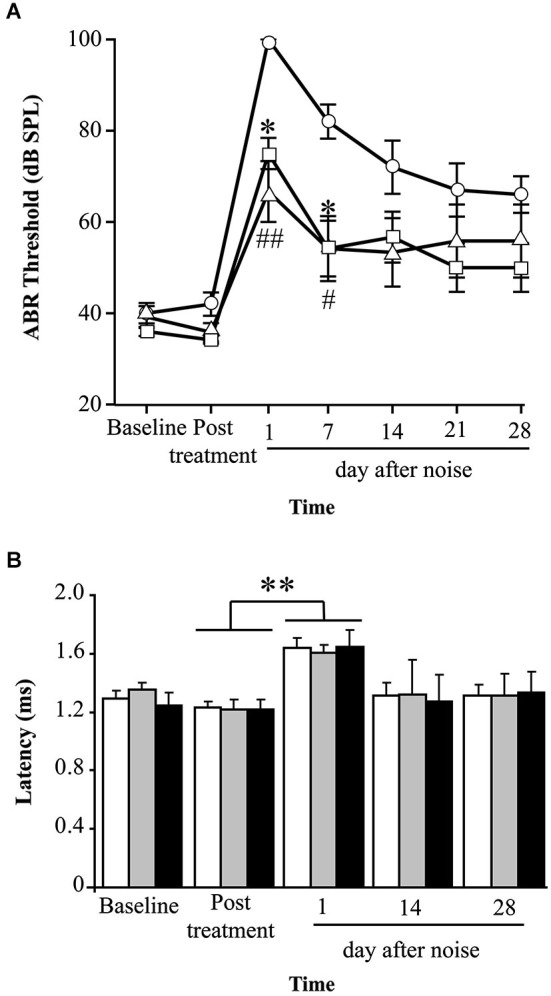
**Evolution of hearing after administration of TGF-β1 inhibitors before noise exposure. (A)** Evolution of ABR thresholds (mean ± SEM, in dB SPL) in MF1 mice (*n* = 6 per group) treated with TGF-β1 inhibitors P17 or P144 at 2.5 mg/kg/24 h/15 days, or saline, and exposed to 100 dB VS noise overnight. Statistically significant differences (ANOVA with Bonferroni *post hoc* test, *p* ≤ 0.05) were found between P17 (triangle, *p ≤ 0.05) or P144 (square, #*p* ≤ 0.05, ##*p* ≤ 0.01,) and the saline group (circles), 1 and 7 days after exposure. **(B)** The evolution of peak I latency (mean ± SEM, in ms) in P17 (black bars), P144 (gray bars) and saline (white bars) treated mice showed a similar pattern. A statistically significant increase in peak I latency was observed 1 day after noise exposure in the three groups (paired *T* test compared to post-treatment values, **p ≤ 0.01).

Mice treated with P17, P144 or saline showed a similar ABR temporal pattern in response to noise; a statistically significant increase in ABR thresholds was observed 1 day after noise exposure when compared to baseline values in the three groups (paired *T* test, *p* ≤ 0.001), which was followed by a gradual decrease during the first 2 weeks and permanent threshold shifts from this moment on (Figure 2A). Compared to saline treatment, systemic injection of P144 and P17 attenuated the threshold shift that occurred in the first day after noise damage, and ameliorated hearing recovery in the following weeks. The preventive effect was more evident in MF1 than in C57 mice, the former showing statistically significant differences 1 and 7 days after noise with both peptides compared to saline (Figure 2A). Non-exposed mice of both strains did not show significant changes of their ABR thresholds over the time (data not shown).

Mice exposed to noise also showed an early statistically significant increase in ABR latencies and a decrease in peak amplitudes, especially in those corresponding to cochlea and spiral ganglion (peak I), and then a recovery of baseline values in the following weeks (Figure 2B). Our results showed that mice treated with TGF-β1 inhibitor peptides P17 and P144 exhibit a similar time-course of peak I latency and amplitude after noise, compared to saline treatment, suggesting that these molecules have a limited effect on nerve conduction.

**Figure 3:**
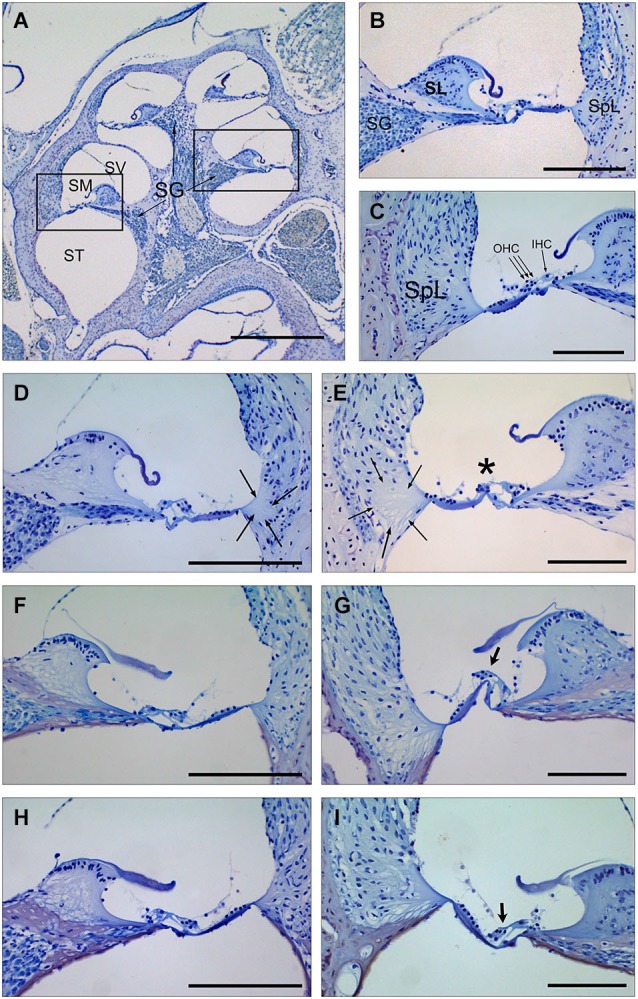
**Cochlear morphology in mice treated with TGF-β1 inhibitors before being exposed to noise. (A–C)** Midmodiolar section **(A)** and detail of the scala media from middle **(B)** and basal **(C)** turns of the cochlea in a control non-exposed mouse, showing normal cytoarchitecture of spiral limbus (SL), spiral ligament (SpL) and organ of Corti with the outer and inner hair cells **(D–I)** Details of the spiral limbus, spiral ligament and organ of Corti from a representative MF1 mouse pre-treated with saline **(D,E)**, P17 **(F,G)** or P144 **(H,I)** before noise exposure. Mice treated with saline showed loss of fibrocyte cells in the spiral ligament (arrows) in the basal and middle turns of the cochlea and outer hair cells in the organ of Corti (asterisk) in the basal turn. In contrast, mice receiving TGF-β1 inhibitors presented preserved cochlear structures, with the presence of OHC even in the basal turn (arrows in **G**,**I**). Scale bars: A, K 0.5 mm; **(C–G)**, 100 μm. SG, spiral ganglion; SV, scala vestibule; SM, scala media; ST, scala tympani; SL, spiral limbus; SpL, spiral ligament; OHC, outer hair cells; IHC, inner hair cells.

Cochlear samples were taken at the end of the experiment (4 weeks after noise exposure) and processed for histological evaluation. Typical morphological alterations were observed in cochlear sections, including changes in stria vascularis, spiral limbus, spiral ligament and organ of Corti, although specific patterns of permanent damage were observed depending on the mouse strain and cochlear region, as described previously (Ohlemiller and Gagnon, 2007; Ohlemiller et al., 2011). In the MF1 strain, mice exposed to noise and treated with saline presented mainly a severe loss of spiral ligament fibrocytes from basal to medium cochlear areas but also a collapsed organ of Corti with lack of outer hair cells in the basal turn. Treatment with TGF-β1 inhibitor peptides P17 and P144 reduced cellular loss in the spiral ligament and favored maintenance of outer hair cells in the organ of Corti, even in basal regions (Figure 3).

### Treatment with P17 and P144 Improves Functional and Morphological Alterations After Noise Exposure

Once the beneficial effect of TGF-β1 inhibitors in the prevention of NIHL was confirmed, we explored their therapeutic properties when administered after noise damage in C57 and CBA mouse strains. C57 mice were exposed to VS^2–20^ noise at 100, 110 or 120 dB SPL, and then treated with TGF-β1 inhibitors or saline. Cochlear samples were taken 1 and 3 days after noise for protein level evaluation, and also at the end the experiment (28 days after exposure) for morphological studies. In parallel, control non-exposed animals were also evaluated.

**Figure 4:**
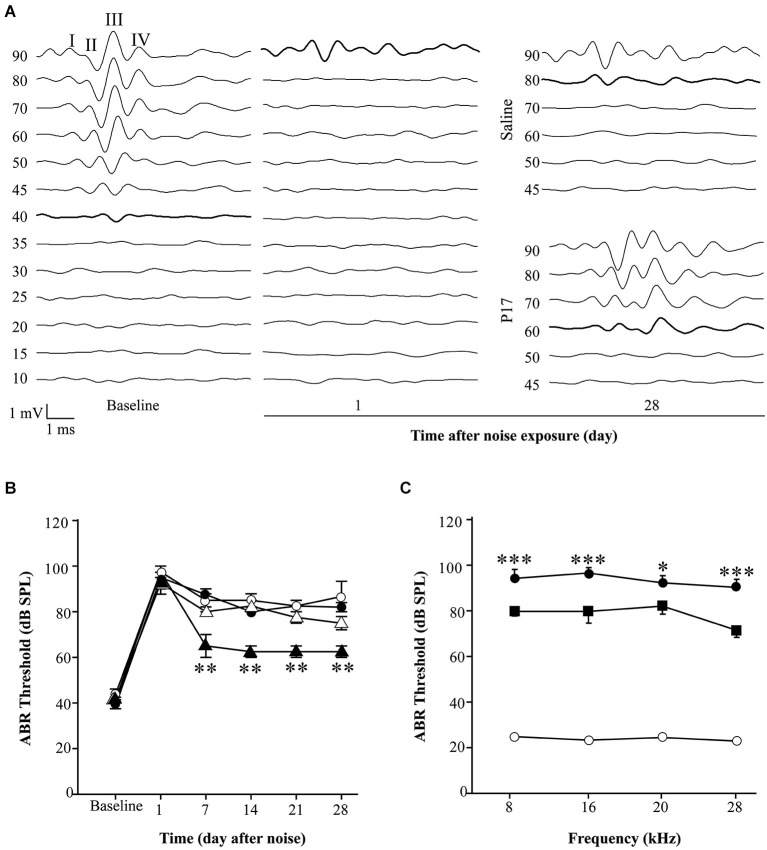
**Administration of TGF-β1 inhibitors after noise exposure. (A)** Representative ABR waveforms in response to click stimulus in C57 exposed to VS^2–20^ noise at 110 dB SPL for 30 min and then treated with TGF-β1 inhibitor P17 at 2.5 mg/kg/12 h or saline for 15 days. Bold lines indicate the ABR threshold. **(B)** Evolution of click ABR thresholds (mean ± SEM, in dB SPL) in C57 mice exposed to VS^2–20^ noise at 110 dB SPL for 30 min and then treated with TGF-β1 inhibitor P17 (triangles) at 2.5 mg/kg or saline (circles) at 0.1 ml/10 g once (white symbols) or twice (black symbols) daily for 15 days (*n* = 6 per group). Statistically significant differences (paired *T* test, **p ≤ 0.01) were found between mice treated with P17 at 2.5 mg/kg/12 h and saline treated mice. Similar results were observed with P144 (data not shown). **(C)** Audiogram of CBA mice treated with P144 (black squares) at 10 mg/kg/day for 15 days after noise exposure (VS^2–20^ at 110 dB SPL, 30 min) showed statistically significant differences (paired *T* test, *p ≤ 0.05, ***p ≤ 0.001) at the end of the treatment compared to saline treated mice (black circles). Non-exposed mice (white circles) maintained normal hearing thresholds throughout the study. Similar results were observed with P17 treatment (data not shown).

Initial experiments using the same dosage as in pre-treatment assays (2.5 mg/kg/24 h) for 15 or even 28 days after noise exposure, did not show statistically significant differences between drug-treated and saline-treated (0.1 ml/10 g/24 h) experimental groups (data not shown). This dose is in the lower range of the reported drug efficiency (Arribillaga et al., 2011) and both molecules have a short life span (Ezquerro et al., 2003), therefore the daily dose was doubled to 2.5 mg/kg/12 h and administered for 15 days. Even under these conditions, P17, P144 and saline treated mice showed similar ABR thresholds after exposure to 120 dB SPL noise. This result indicates that TGF-β1 inhibitors were not able to restore hearing function after an acoustic trauma (data not shown). However, in non-traumatic exposures to noise intensities under 120 dB SPL, both inhibitors clearly improved the time course of hearing loss beginning the first week after noise-exposure. The favorable effect on the evolution of ABR thresholds was more evident in mice treated with P17 (Figures 4A,B), whereas P144 showed a subtle therapeutic effect, possibly because of its poorer pharmacokinetic profile. Statistically significant differences in ABR thresholds, compared to saline-treated mice, were observed when P144 doses were increased to the maximum recommended dose of 10 mg/kg/24 h for 15 days (Figure 4C). Thus, TGF-β1 inhibitors showed a dose-dependent therapeutic effect on NIHL.

**Figure 5:**
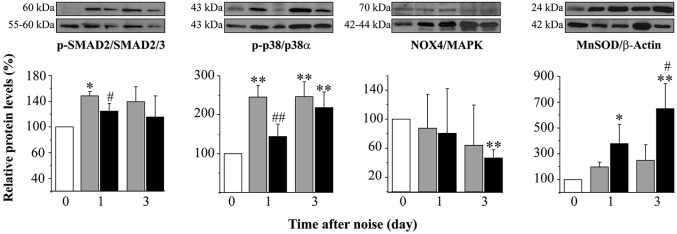
**Protein levels after noise exposure and treatment with TGF-β1 inhibitor**. Time course of phospo-p38/p38α, phospho-SMAD2/SMAD2/3, NOX-4/MAPK and MnSOD/β-actin ratios relative (%) to non-exposed mice (white bars), in cochlear samples of noise-exposed (VS^2–20^ at 110 dB SPL) CBA mice (*n* = 3 mice for each determination) and treated with TGF-β1 inhibitor P144 at 10 mg/kg/24 h/15 days (black) or saline (gray). Representative blots are shown. Data are shown as mean ± SD. Statistically significant differences were found in p-p38, NOX4 and MnSOD levels after noise exposure compared to values in non-exposed mice (paired *T* test, *p ≤ 0.05; **p ≤ 0.01). Noise induced statistically significant increases in p-p38, p-SMAD2 and MnSOD ratios compared to non-exposed mice (paired *T* test, *p ≤ 0.05; **p ≤ 0.01). Treatment with P144 showed significantly lower levels of p-p38α and p-SMAD2 and higher levels of MnSOD compared to saline-treated mice (paired *T* test, #*p* ≤ 0.05; ##*p* ≤ 0.01).

As aforementioned, mice exposed to noise showed an early increase in p-p38α, p-SMAD2 and MnSOD protein levels in the cochlea regardless of treatment, in comparison to non-exposed control mice. In agreement with functional results, mice treated with TGF-β1 inhibitors showed significantly lower values of p-p38α and p-SMAD2 1 day after damage and a higher MnSOD level 3 days after insult when compared to saline-treated mice, suggesting that these peptides favored an anti-inflammatory and antioxidant state throughout TGF-β1 signalling inhibition (Figure 5).

Cochlear samples were taken for gross histology evaluation 4 weeks after noise exposure. Similarly to pre-treatment assays, treatment with TGF-β1 inhibitor peptides P17 and P144 attenuated the morphological alterations induced by VS^2–20^ noise compared to saline treated animals (data not shown). Differences were especially observed in the lateral wall structures and in the organ of Corti, depending on the mouse strain. In CBA mice, TGF-β1 inhibitors reduced the degenerative changes in the spiral ligament and the loss of fibrocytes, whereas in C57 mice, the protective effect was more evident in the organ of Corti, with maintenance of outer hair cells even in basal regions (data not shown).

## Discussion

According to WHO, NIHL is an important medical concern in developed countries and currently efforts are being focused on preventing excessive noise exposure and on treating hearing loss (updated February 2014).^2^

Noise exposure induces a series of well-characterized morphological and functional changes in the cochlea, depending especially on the noise level, frequency, duration and temporal pattern (Wang et al., 2002; Hirose and Liberman, 2003; Ohlemiller, 2006; Park et al., 2013). The principal pathogenic mechanisms in NIHL include oxidative stress (Henderson et al., 2006) and inflammatory response, with an early local expression of cytokines and recruitment of immune cells (Tan et al., 2013), As occurred in ototoxicity, molecules with antioxidant and anti-inflammatory actions are being tested as potential otoprotective compounds (Bas et al., 2012; Fetoni et al., 2013).

The participation of the proinflammatory cytokines TNF-α, IL-1β and IL-6 in the cochlea after noise exposure has been reported (Ichimiya et al., 2000; Satoh et al., 2002; Fujioka et al., 2006; Tahera et al., 2006; Nakamoto et al., 2012; Tan et al., 2013), but the role of TGF-β is still undetermined. TGF-β family factors are key regulators of the immune and inflammatory response in several processes (reviewed in Mantel and Schmidt-Weber, 2011). In fact, an early increase in TGF-β expression in the inner ear has also been described in ototoxicity, immuno-mediated hearing loss and chronic otitis media (Satoh et al., 2006; Wissel et al., 2006; Ghaheri et al., 2007).

In this work we show that in the first 24 h after noise exposure there is an increase in *Tgfb1* and *TgfbR1* cochlear gene expression, concomitantly with a decrease in *Tgfb2* and *TgfbR2*. These results point to the participation of TGF-β1 in the cochlear inflammatory response to noise injury, and suggest that TGF-β1 could play a role in the initial inflammatory phase. Thus, additional experiments from the group in a mice model of NIHL found a statistically significant 3-fold change in *Tgfb1* expression 4 h after noise challenge, compared to non-exposed animals (Celaya et al., in preparation). Similarly, up-regulation of the proinflammatory cytokines TNFα, IL-1β and IL-6 has been described to occur rapidly after noise exposure, reaching maximum level 3–6 h after noise exposure and maintaining high levels during the following 24–48 h (Fujioka et al., 2006; Tornabene et al., 2006). This quick inflammatory response presumably originates in resident cells, including bone marrow-derived macrophages, spiral ganglion cells and spiral ligament fibrocytes, but also from recruited immune cells. Thus, Satoh et al. found an increase in TGF-β1 immunostaining in the infiltrated inflammatory cells 3 h after the injection of keyhole limpet hemocyanin, which caused an exacerbated immune response in the mouse cochlea. These high levels of TGF-β1 persisted for 48 h and reverted to normal after 7 days as the response resolves (Satoh et al., 2006). TGF-β has been shown to possess dual actions in the inflammatory response to damage, with both pro and anti-inflammatory roles (Kawamoto et al., 2003; Sanjabi et al., 2009). Therefore, we can speculate that the observed initial release of the factor in the cochlea forms part of the early proinflammatory phase of cochlear response to noise damage. Indeed, our data show that its inhibition has an overall protective effect.

In our experiments, noise also induced an activation of the proinflammatory p38α MAPK in the cochlea. We studied the ratio of phosphorylated to total kinase levels as an index of activity in cochlear protein extracts, and we found an increase at 24 h and 2 weeks after VS^2–20^ noise exposure in the saline-treated mouse group. The activation of p38α after noise damage and its correlation with temporal and permanent threshold shifts have been previously reported in the cochlea of chinchilla (Jamesdaniel et al., 2011) and mouse (Meltser et al., 2010; Maeda et al., 2013).

We also confirmed elevated NOX-4 levels in the cochlea 1–3 days after noise exposure. NADPH oxidases (NOX) are enzymes that transport electrons across the plasma membrane and constitute an important source of superoxide radicals. Excessive production of superoxide increases levels of reactive oxygen and nitrogen species which could damage DNA and disrupt lipid and protein molecules leading to cell death by apoptosis (Henderson et al., 2006). Oxidative stress is a common pathogenic mechanism in cochlear damage secondary to noise, ototoxic drugs and aging. Thus, an increase in the expression of NOX-1 and NOX-4 isoforms and their regulatory subunits has been described in fibrocytes, epithelial cells and neurons from mice treated with cisplatin (Kim et al., 2010). In noise-exposed rats, it has been observed an up-regulation of NOX-1 and DUOX2 whereas NOX-3 was down-regulated (Vlajkovic et al., 2013).

In parallel to NOX-4, our data also confirmed an elevation of MnSOD protein level in the cochlea after noise damage exposure. Superoxide dismutases are key antioxidant enzymes directed toward the scavenging of free radicals to maintain the oxidative balance, and low levels are associated with an impaired response to cochlear damage in mice and rats (Keithley et al., 2005; Ying and Balaban, 2009). In addition, polymorphisms in the human *SOD2* gene that codifies for MnSOD enzyme, have been related to NIHL predisposition (Fortunato et al., 2004; Liu et al., 2010), suggesting that this enzyme is critical during the cochlear response to noise injury.

Taken together, our data confirm that inflammation and oxidative stress are central events in the physiopathology of NIHL and show that TGF-β1 participates in the early phases. Therefore, blocking of this proinflammatory cytokine could be useful in ameliorating pathological changes in the cochlea after noise insult. In this study we tested the therapeutic effect of TGF-β1 inhibitors P17 and P144 administered systemically both before and after noise exposure to explore their potential in prevention and repair situations, respectively. These peptides have demonstrated a strong TGF-β inhibitory effect in animal models of liver and pulmonary fibrosis and hypertension (Ezquerro et al., 2003; Arribillaga et al., 2011; Baltanás et al., 2013). In addition P144 has completed phase I and II clinical trial studies (NCT00656825, NCT00574613 from http://clinicaltrials.gov/ct2/home) and it has been designated as an orphan drug.

Systemic pre-treatment with P17 or P144 significantly reduced the temporal threshold shift observed 1 day after noise exposure. However, when the same dose of TGF-β1 inhibitors were administered after noise damage, the therapeutic effect was observed only after 1 week of treatment. Intraperitoneal administration of drugs could achieve rapidly high cochlear levels (Rivera et al., 2012). For example, steroids reach the highest concentration in the mouse cochlea only 25 min after injection (Kanzaki et al., 2012). On the other hand, up-regulation of proinflammatory cytokines, including TGF-β1, occurs in the first 3–6 h after noise exposure (Fujioka et al., 2006; Tornabene et al., 2006; Celaya et al., in preparation). Therefore, to reach effective local levels in the acute phase of noise-induced inflammation, drug delivery should be performed before or immediately after exposure. We hypothesize that this is the reason why a similar dose of TGF-β1 inhibitors (2.5 mg/kg/day/15 days) was more effective if administered prior to noise exposure rather than 1 day afterwards. The therapeutic effect of TGF-β1 inhibitors administered after noise exposure depended on the noise level and also on the peptide dosages. Thus, P17 and P144 were not able to improve the functional and structural damage that occurred after acoustic trauma with noise levels of 120 dB SPL. However, dose-dependent beneficial effects could be observed when these peptides were administered after exposure to non-traumatic 100 or 110 dB SPL noise. P17 induced a better recovery of ABR thresholds when administered at 5 mg/kg daily for 15 consecutive days, whereas P144 required 10 mg/kg daily dose to achieve similar effectiveness. This result might possibly reflect the pharmacological characteristics of these peptides, since P144 has a poorer solubility in saline than P17 (Ezquerro et al., 2003).

Concomitantly to functional improvement, mice treated with TGF-β1 inhibitors presented more favorable antioxidant and anti-inflammatory profiles than saline-treated mice. Thus, a significant decrease in the activation of the stress-related kinase p38α was observed in mice treated with TGF-β1 inhibitors 1 day after noise exposure compared to saline-treated mice, suggesting that these peptides contribute to decrease the activity of proinflammatory pathways after noise injury. Oxidative imbalance by excessive formation of free radical species is one of the main pathological mechanisms in NIHL (Henderson et al., 2006). Treatment of NIHL with TGF-β1 inhibitors also induces an increase in the levels of MnSOD compared to saline controls, therefore contributing to the scavenging of free radicals and recovery of the oxidative balance. Both the decrease in p38α and the increase in MnSOD activities should provide mice treated with TGF-β1 inhibitors a better resistance to NIHL.

The morphological substrate for this therapeutic effect seems to be the cochlear lateral wall, since noise-exposed mice treated with TGF-β1 inhibitors showed reduced pathological alterations in the spiral ligament when compared to those receiving saline. It has been well documented that noise exposure results in disruption of the lateral wall and causes strial edema and apoptosis of fibrocytes of the spiral ligament (Hirose and Liberman, 2003). The lateral wall is a central structure in the modulation of cochlear inflammatory and immune responses to insult, with resident macrophages and also fibrocytes secreting cytokines that modify the permeability of the blood-labyrinth barrier and attract immune cells from the vasculature. It is likely that TGF-β1 levels are increased in the spiral ligament after noise exposure, therefore acting as a proinflammatory cytokine. In addition, it has been reported that mononuclear phagocytes migrate into the murine cochlea after acoustic trauma (Hirose et al., 2005) and that infiltrated inflammatory cells express TGF-β after antigen challenge (Satoh et al., 2006). Thus blocking this factor could be a good strategy for ameliorating noise-induced cochlear changes.

On the other hand, it is known that TGF-β1 stimulates the production of collagen fibers in fibrocytes and that excessive collagen synthesis could be deleterious. In this context, the inhibition of TGF-β1 has shown important effects on fibrotic diseases, including hepatic (Ezquerro et al., 2003), pulmonary (Arribillaga et al., 2011), myocardial (Hermida et al., 2009), skin (Santiago et al., 2005) and periprosthetic fibrosis (San-Martin et al., 2010). Therefore, reduction of excessive collagen formation in the spiral ligament after noise exposure could be a secondary beneficial action of TGF-β1 inhibitors in the cochlea.

In summary, in this study we show that TGF-β1 plays a central role in cochlear response to excessive noise exposure and that treatment with TGF-β1 inhibitors ameliorates NIHL.

## Authors and Contributors

SM-C, acquisition of data, analysis and interpretation of data, drafting and revising the article, final approval of the version to be published. LR-dlR, acquisition of data, analysis and interpretation of data, drafting the article. JC, analysis and interpretation of data, drafting the article. GC, acquisition of data. TR, analysis and interpretation of data, revising the article, final approval of the version to be published. IV-N, design of the experiments, analysis and interpretation of data, revising the article, final approval of the version to be published.

## Conflict of Interest Statement

The authors declare that the research was conducted in the absence of any commercial or financial relationships that could be construed as a potential conflict of interest.

## Footnotes

^1^http://www.integromics.com/genomics-data-analysis/pcr-analysis

^2^http://www.who.int/mediacentre/factsheets/fs300/en/

## Abbreviations

ABR, Auditory brainstem response; NIHL, Noise-induced hearing loss; OHC, Outer hair cells; TGF-β, Transforming growth factor beta; VS, Violet swept sine.
