# Corrigendum: Transforming growth factor β1 inhibition protects from noise-induced hearing loss

**DOI:** 10.3389/fnagi.2015.00072

**Published:** 2015-05-08

**Authors:** Silvia Murillo-Cuesta, Lourdes Rodríguez-de la Rosa, Julio Contreras, Adelaida M. Celaya, Guadalupe Camarero, Teresa Rivera, Isabel Varela-Nieto

**Affiliations:** ^1^Institute of Biomedical Research “Alberto Sols” (IIBM), Spanish National Research Council–Autonomous University of Madrid (CSIC-UAM) Madrid, Spain; ^2^Centre for Biomedical Network Research on Rare Diseases (CIBERER), Institute of Health Carlos III (ISCIII) Madrid, Spain; ^3^Hospital La Paz Institute for Health Research (IdiPAZ) Madrid, Spain; ^4^Veterinary Faculty, Complutense University of Madrid Madrid, Spain; ^5^Príncipe de Asturias University Hospital, University of Alcalá, Alcalá de Henares Madrid, Spain

**Keywords:** cochlear injury, inflammation, noise-induced hearing loss, protection, TGF-ß

Unfortunately, the second author affiliation in the originally published article was incorrectly stated as being “Centre for Biomedical Network Research (CIBER), Institute of Health Carlos III (ISCIII), Madrid, Spain.”

The correct author's affiliation is “Centre for Biomedical Network Research on Rare Diseases (CIBERER), Institute of Health Carlos III (ISCIII), Madrid, Spain.”

## Conflict of interest statement

The authors declare that the research was conducted in the absence of any commercial or financial relationships that could be construed as a potential conflict of interest.

